# Advances in Respiratory Medicine (ARM)—Past, Present, Future

**DOI:** 10.3390/arm90040040

**Published:** 2022-08-08

**Authors:** Adam Barczyk

**Affiliations:** Department of Pneumonology, School of Medicine in Katowice, Medical University of Silesia in Katowice, 40-635 Katowice, Poland; adagne@icloud.com

## 1. Past

Advances in Respiratory Medicine (ARM) is the journal of the Polish Respiratory Society. As with the Society itself, it grew out of phthisiatry. The first issue of the journal was published in 1909 under the Polish title Gruźlica (Tuberculosis). Interestingly, the Society itself was founded much later, in 1934, as Polskie Towarzystwo Badań Naukowych nad Gruźlicą (the Polish Society for Scientific Research on Tuberculosis). Therefore, tuberculosis is at the root of contemporary pulmonology, and probably not only in Poland. Why? At that time, tuberculosis was one of the most important diseases. In 1909, tuberculosis was still the leading cause of death in the US (it was replaced shortly thereafter by heart disease). (1) In Poland, tuberculosis was the most important cause of death for much longer, until the 1950s, which was partly a result of the enormous destruction and poverty caused by World War II. (2) The name of the journal and the society therefore corresponded to the main medical problem of the time. Over time, both the journal and the Society were renamed, gradually losing their nomenclature to tuberculosis. In 1976, the journal changed its name to Pneumonologia Polska (Polish Pneumonology), but a similar movement on the side of the Society took place much later, because it was not until 2006 that the Society lost tuberculosis in its name, changing its name to Polskie Towarzystwo Chorób Płuc—PTChP (the Polish Respiratory Society—PRS).

For many years, the journal published articles in Polish, written by Polish authors for Polish phthisiatrists, and then pulmonologists. In addition, the journal published a mixed set of articles to combine two functions: a research journal (mainly original articles) and an educational journal (mainly review articles and case reports). About 15 years ago, the journal began to change. The changes resulted from several factors. The first was the organizational change of the Society itself. Not only has the name changed, but the entire structure of the organization has changed. At the same time, Poland was changing rapidly. Gradually, new requirements regarding the quality of scientific achievements emerged, which forced the necessity to start changes to the journals. Additionally, the expectations of the Society’s members changed. Those of us who worked in scientific institutions expected, above all, articles of high scientific quality, with an obvious necessity to publish in English. This, however, did not satisfy the majority of the Society’s members, working only as medical practitioners, who, in turn, expected further articles in Polish, preferably of a purely educational nature. As a consequence, 10 years ago, PTChP (PRS) began the process to publish two different journals, the previous one, which changed their name to the English-language one—Advances in Respiratory Medicine, and a new educational journal with the old name Pneumonologia Polska. For the last 8 years, one publisher and one editorial team have served the two journals above. ARM has undergone a number of changes in the last few years, as a result of which, all articles are published only in English and online; the vast majority of articles come from abroad; and the journal is indexed in scientific databases (Scopus, Index Medicus, MEDLINE, PubMed and Emerging Sources Citation Index).

## 2. Present

The latest changes in the journal began last year with the decision of the previous ARM editorial board to focus their efforts only on the educational journal—Pneumonologia Polska. The PTChP (PRS) board appointed a new editorial board of the journal, and the function of the Editor-in-Chief was taken over by the undersigned. The new editorial team started to work on 1 October 2021. What problems did we encounter? First of all, the journal, despite 15 years of attempts, was not able to enter the list of journals with an Impact Factor. Secondly—for Polish authors, the journal has ceased to be attractive as a result of science reforms in Poland in recent years, which resulted in an almost complete lack of original articles from Poland. Thirdly, a very large number of articles from abroad was sent to the ARM, but their quality was low. Fourth, there was a growing problem with the reviewers, as foreign authors mostly refused to review, so this obligation fell on the vast majority of Polish pulmonologists. Fifthly, the time from submitting the article to its publication was becoming longer and longer, which was partly a result of the problem with the reviewers. These are just a few of the many problems we found. We started a series of activities, the most important of which was the search for a new publisher who would help us achieve the most important goal, namely a quick increase in the scientific quality of the published articles, so that it would become realistic to obtain an Impact Factor for the journal. After many months of negotiations, PTChP (PRS) signed a contract for the publishing of ARM by MDPI—an international publishing company, a leader in publishing open access scientific articles. As of 1 July 2022, ARM is issued by MDPI.

## 3. Future

Our goal for the next 5 years is to quickly improve the scientific quality of the published articles so that it becomes realistic to achieve an Impact Factor at the end of this period. To make it possible, we will look for the best possible authors writing the best scientific papers. Thanks to the cooperation with MDPI, many of our problems are already improving. Let me give two examples: better access to a larger group of foreign reviewers and significantly shortening the time from submitting an article to ARM to its final approval. We plan to look for new ARM editors from among active authors and reviewers. I firmly believe that a new and brilliant period in the history of the journal has begun.

